# Diverse impacts of female chromosomal polymorphisms on assisted reproduction outcomes: a retrospective cohort study

**DOI:** 10.1186/s12884-024-06532-w

**Published:** 2024-04-27

**Authors:** Yongjie Lu, Tian Tian, Lixue Chen, Liying Yan, Liang Chang, Jie Qiao

**Affiliations:** 1 State Key Laboratory of Female Fertility Promotion, Center for Reproductive Medicine, Department of Obstetrics and Gynecology, Peking University Third Hospital, Beijing, 100191 China; 2 National Clinical Research Center for Obstetrics and Gynecology, Peking University Third Hospital, Beijing, 100191 China; 3 Key Laboratory of Assisted Reproduction (Peking University), Ministry of Education, Beijing, 100191 China; 4 Beijing Key Laboratory of Reproductive Endocrinology and Assisted Reproductive Technology, Beijing, 100191 China; 5 National Clinical Key Specialty Construction Program, P. R. China 2023, Beijing, 100191 China

**Keywords:** Assisted reproduction, Chromosomal abnormalities, Infertility, IVF/ICSI outcome, Oocyte quality

## Abstract

**Background:**

The effects of female chromosomal polymorphisms (FCPs) on various aspects of reproductive health have been investigated, yet the findings are frequently inconsistent. This study aims to clarify the role of FCPs on the outcomes of in vitro fertilization (IVF) and intracytoplasmic sperm injection (ICSI).

**Methods:**

This retrospective cohort study comprised 951 couples with FCPs and 10,788 couples with normal karyotypes who underwent IVF/ICSI treatment at Peking University Third Hospital between 2015 and 2021. The exposure was FCPs. The embryological outcomes and clinical outcomes were compared.

**Results:**

The FCPs, as a whole, compromised the oocyte maturation rate (76.0% vs. 78.8%, *P* = 0.008), while they did not adversely affect other IVF/ICSI outcomes. Further detailed analyses showed that every type of FCPs contributed to the lower oocyte maturation rate, particularly the rare FCPs (69.0% vs. 78.8%, *P* = 0.008). The female qh + was associated with a higher normal fertilization rate (63.0% vs. 59.2%, adjusted *P* = 0.022), a higher clinical pregnancy rate (37.0% vs. 30.7%, adjusted *P* = 0.048), and a higher live birth rate (27.0% vs.19.0%, adjusted *P* = 0.003) in couples undergoing IVF. Conversely, in couples undergoing ICSI, female qh + was found to be related to a lower normal fertilization rate (58.8% vs. 63.8%, *P* = 0.032), a comparable clinical pregnancy rate (25.7% vs. 30.9%, *P* = 0.289), and a comparable live birth rate (19.8% vs. 19.2%, *P* = 0.880) compared to the control group. Additionally, an increased risk of preterm birth was observed in women undergoing IVF with multiple polymorphisms (62.5% vs. 16.9%, adjusted *P* <  0.001) and in women undergoing ICSI with pstk+ (36.4% vs. 15.4%, *P* = 0.036).

**Conclusions:**

Our research unravels the diverse impacts of various FCPs on IVF/ICSI outcomes, highlighting the detrimental effects of FCPs on oocyte maturation and the risk of preterm birth.

**Supplementary Information:**

The online version contains supplementary material available at 10.1186/s12884-024-06532-w.

## Background

Chromosomal polymorphisms refer to the variations in the size or structure of heterochromatin regions. The common chromosomal polymorphisms include increased lengths of pericentric heterochromatin (qh+) on chromosomes 1, 9, and 16, increased stalks (pstk+) on chromosomes 13, 14, 15, 21, and 22, and inversion within the pericentric heterochromatin of chromosome 9 (inv(9)(p12q13), abbreviated as inv(9) in this article). Other less common polymorphisms include double or increased satellites (pss or ps+) on chromosomes 13, 14, 15, 21, and 22, inversion within pericentric heterochromatin of chromosomes 1, 2, 3, 10, and 16, etc. [1]. The females do not involve Y chromosome polymorphisms.

Despite being generally regarded as harmless, chromosomal polymorphisms were more readily detected in individuals suffering from reproductive disorders and were linked with adverse outcomes of assisted reproduction [2–8]. In addition, male chromosomal polymorphisms (MCPs) and female chromosomal polymorphisms (FCPs) were reported to exert distinct impacts on the outcomes of reproductive health [9–12], necessitating separate investigations into MCPs and FCPs. This research was dedicated to examining the impacts of FCPs.

Compared with couples with normal karyotypes, couples with FCPs exhibited lower fertilization rates (24 couples with female D/G group polymorphisms or 37 couples with female inv(9) vs. 1088 normal couples in [12]), lower cleavage rates (99 couples with FCPs vs. 400 normal couples in [10], hereafter abbreviated as 99 vs. 400), lower embryo quality (86 vs. 214 in [13]), higher miscarriage rates (101 vs. 2704, and 81 vs. 2135 in [2]), and higher preterm birth rates (101 vs. 2704, 81 vs. 2135, 163 vs. 2188, 45 vs. 921, and 33 vs. 997 in [2]). However, the results from other studies refuted these findings (82 vs. 1402 in [14]; 150 vs. 448 in [15]; 262 vs. 9713, and 311 vs. 10,858 in [16]). These conflicting conclusions, which derived from studies where the number of couples with FCPs was small and the FCPs were usually analyzed as a whole, are anxiety-provoking for patients and confusing for clinicians.

Here, we comprehensively investigated the impacts of various FCPs on IVF/ICSI outcomes in up to 951 couples with FCPs and 10,788 control couples with normal karyotypes and revealed the exact associations of various types of FCPs with different assisted reproduction outcomes.

## Methods

### Study participants and ethical approval

This study is a retrospective cohort study. A total of 11,739 infertile couples who received IVF/ICSI treatment and karyotyping at Peking University Third Hospital between 2015 and 2021 were included. The control group consisted of couples with normal karyotypes and the FCP group included couples in which the female partner carried chromosomal polymorphism(s) and the male partner had a normal karyotype. The following couples were excluded, (i) couples with chromosome aberration, mosaic karyotype, or monogenic disease, (ii) couples in which the male partner carried chromosomal polymorphism(s), (iii) couples received gamete donation. The study incorporated the initial ovarian stimulation cycle and the initial corresponding embryo transfer cycle (fresh or frozen) for each couple. The Peking University Third Hospital Medical Science Research Ethics Committee granted ethical approval for this study (IRB00006761-M2023384). Informed consent exemptions were approved by the ethics committees due to the retrospective nature of this study.

### Karyotype analysis and classification of chromosomal polymorphisms

G-banding karyotype with a resolution of 400–550 bands was performed according to standard procedures. At least 20 metaphases were examined for each participant. The karyotyping results were reviewed by two experienced cytogeneticists independently. In accordance with the International System for Chromosome Nomenclature 2013 [1], chromosomal polymorphisms were reported when the heterochromatin exhibited a size greater than twice that of their homologous counterparts. The qh+/− represents the variations in the pericentric heterochromatin of chromosomes 1, 9, and 16. The pstk+/−, pss, and ps + represent the variations of the stalk and satellites on the short arms of the acrocentric chromosomes 13, 14, 15, 21, and 22. The inv(9) is the inversion within the pericentric heterochromatin of chromosome 9. Other inversions within the pericentric heterochromatin of chromosomes 1, 2, 3, 10, and 16 are also regarded as polymorphisms. The cenh+ represents an increase in the size of centromeric heterochromatin. Fig. S1 displayed representative images of chromosomal polymorphisms.

### Variables and outcomes

The age of females and males, body mass index (BMI) of females and males, basal endocrine level (follicle stimulating hormone (FSH) and E2), antral follicle count (AFC), type (primary or secondary) and cause (tubal factor, diminished ovarian reserve, polycystic ovary syndrome (PCOS), endometriosis, other maternal factors, and paternal factor) of infertility, and various treatment parameters (methods of stimulation, fertilization, and embryo transfer; stage and number of the transferred embryo) were collected as baseline data.

Definitions and assessments of the IVF/ICSI embryological outcomes and clinical outcomes were performed as previously described [17]. Briefly, 36 hours after the trigger of human chorionic gonadotropin (HCG), the oocytes were retrieved. The presence of a first polar body (PB) indicated mature oocytes (MII oocytes), and the oocyte maturation rate was the proportion of MII oocytes relative to the total number of retrieved oocytes. Normal fertilization was identified by the observation of a second polar body (PB) and two pronuclei (PN) within 16 to 18 hours following insemination. The normal fertilization rate was calculated as the proportion of oocytes exhibiting normal fertilization to the total number of oocytes inseminated. Embryo quality was evaluated 67 to 69 hours post-insemination (Day 3) based on cell count and cytoplasmic fragmentation extent. The transplantable embryo rate was derived by calculating the ratio of embryos that advanced from the 2PN oocytes and attained a stage of five or more cells with cytoplasmic fragmentation not exceeding 30% on Day 3, to the number of embryos that displayed cleavage on Day 2. For the assessment of clinical outcomes, biochemical pregnancy was defined by a serum β-hCG level of more than 10 IU/L, measured 14 days after the transfer of embryos. Clinical pregnancy was confirmed via ultrasound by observing at least one gestational sac 30 days after the embryo transfer. Following the expert consensus and guidelines from China, miscarriage refers to the loss of a pregnancy before 28 weeks of pregnancy [18]. Preterm birth refers to the parturition before 37 weeks of pregnancy [19]. A live birth was defined as the successful delivery of one or more live neonates. The denominator for calculating the biochemical pregnancy rate, clinical pregnancy rate, and live birth rate was the number of couples receiving embryo transfer, while the denominators for calculating the miscarriage rate and preterm birth rate were couples with clinical pregnancy and those with successful delivery, respectively.

### Statistical analysis

Given that the continuous variables in the present study were non-normally distributed, they were reported as the median with the 25th and 75th percentiles, and their comparisons were conducted using the Mann-Whitney U test or the Kruskal-Wallis test. Categorical variables were presented as the number and percentage, and their comparisons were performed using the Chi-square test or Fisher’s exact test. The significantly different variables (*P* <  0.05) were selected as the confounders for adjustment in the multivariate analyses. Generalized linear regression models with adjustments for potential confounders were employed to compare the embryological outcomes of IVF/ICSI and yielded estimated marginal means (EMMs)(the adjusted means), coefficients, and adjusted *P* values. Log-binomial regression models with adjustments for potential confounders were applied to evaluate the clinical outcomes of IVF/ICSI, and the adjusted risk ratios (aRRs) and adjusted *P* values were obtained. Statistical analyses were performed with SPSS version 29.0 (IBM, Inc.). *P* < 0.05 was considered statistically significant.

## Results

### Baseline characteristics

**Fig. 1:**
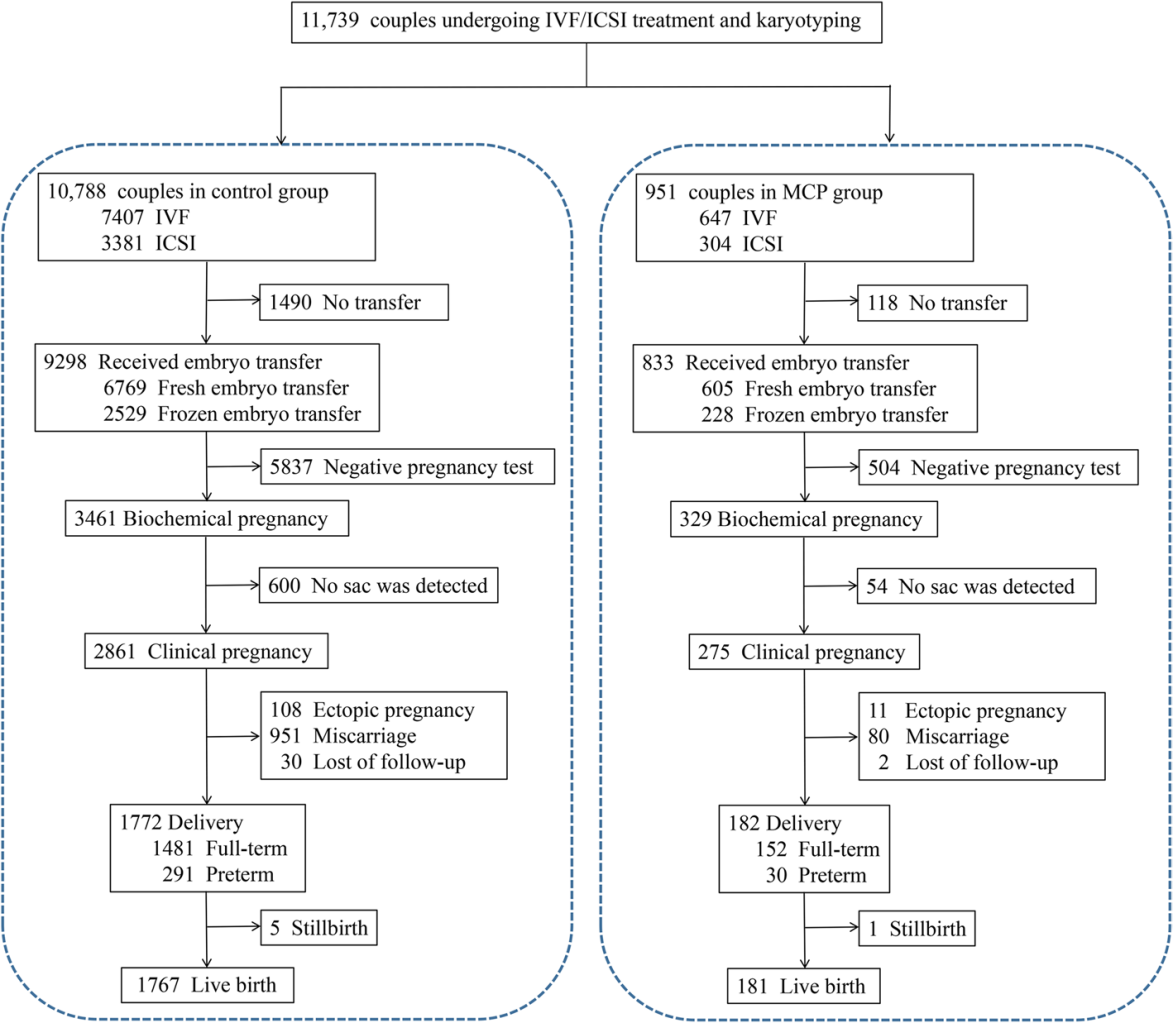
Flow chart of participants at each stage of IVF/ICSI treatment

Figure 1 displayed the distribution of couples at various stages of IVF/ICSI treatment. The FCP group included 951 couples and the control group included 10,788 couples (Table 1). The two groups were comparable in terms of maternal age, paternal age, maternal BMI, paternal BMI, basal FSH level, basal E2 level, AFC, infertility type, tubal factor, PCOS, endometriosis, other maternal factors, stimulation protocol, fertilization type, embryo transfer method, stage of transferred embryo, and number of transferred embryo (Table 1). In the FCP group, the proportions of couples with diminished ovarian reserve (10.7% vs. 13.4%, *P* = 0.020, Table 1) and paternal infertility factor (47.2% vs. 52.1%, *P* = 0.004, Table 1) were lower than those in the control group. These two variables would be adjusted in the following multivariate analysis models. According to the types of polymorphisms, the FCP group was divided into five subgroups: qh + (352, 37.0%), pstk+(307, 32.3%), inv(9)(118, 12.4%), multiple (including couples who carried two or more FCPs)(91, 9.6%), and others (including couples who carried other less common FCPs such as ps+, inv(1)(p13q21), etc.)(83, 8.7%) (Table 1).

**Table 1:** Comparison of baseline characteristics between the control and FCP group

	Control *n* = 10,788	FCP group *n* = 951	*P* value
Maternal age, years	32.0(30.0 ~ 35.0)	32.0(30.0 ~ 35.0)	0.542
Paternal age, years	33.0(30.0 ~ 36.0)	33.0(30.0 ~ 36.0)	0.390
Maternal BMI, kg/m2	22.2(20.2 ~ 24.8)	22.0(20.2 ~ 25.0)	0.786
Paternal BMI, kg/m2	25.1(23.0 ~ 27.7)	25.1(23.0 ~ 27.8)	0.734
Basal FSH level, IU/L	6.3(4.8 ~ 7.9)	6.2(4.9 ~ 7.9)	0.705
Basal E2 level, pmol/L	155.0(114.0 ~ 200.0)	152.0(114.0 ~ 198.0)	0.671
AFC	11.0(8.0 ~ 15.0)	10.0(8.0 ~ 15.0)	0.752
Infertility type			0.653
Primary	6354(58.9)	553(58.1)	
Secondary	4434(41.1)	398(41.9)	
Tubal factor			0.908
No	6770(62.8)	595(62.6)	
Yes	4018(37.2)	356(37.4)	
PCOS			0.312
No	8878(82.3)	795(83.6)	
Yes	1910(17.7)	156(16.4)	
Diminished ovarian reserve			**0.020**
No	9345(86.6)	849(89.3)	
Yes	1443(13.4)	102(10.7)	
Endometriosis			0.747
No	9686(89.8)	857(90.1)	
Yes	1102(10.2)	94(9.9)	
Other maternal factors			0.869
No	8711(80.7)	770(81.0)	
Yes	2077(19.3)	181(19.0)	
Paternal factor			**0.004**
No	5167(47.9)	502(52.8)	
Yes	5621(52.1)	449(47.2)	
Stimulation protocol			0.301
GnRH agonist	3689(34.2)	341(35.9)	
GnRH antagonist	7099(65.8)	610(64.1)	
Fertilization type			0.690
IVF	7407(68.7)	647(68.0)	
ICSI	3381(31.3)	304(32.0)	
Embryo transfer method			0.480
Fresh	6769(62.7)	605(63.6)	
Frozen	2529(23.4)	228(24.0)	
No transfer	1490(13.8)	118(12.4)	
Stage of transferred embryo			0.474
Cleavage stage	8041(86.5)	713(85.6)	
Blastocyst stage	1257(13.5)	120(14.4)	
Number of transferred embryo			0.538
one	1991(21.4)	186(22.3)	
two	7307(78.6)	647(77.7)	
Type of FCPs			
qh+	NA	352(37.0)	
pstk+	NA	307(32.3)	
inv(9)	NA	118(12.4)	
multiple	NA	91(9.6)	
others	NA	83(8.7)	

*FCP* female chromosomal polymorphism, *BMI* body mass index, *FSH* follicle stimulating hormone, *AF*, antral follicle count, *PCOS* polycystic ovarian syndrome, *IVF* in vitro fertilization, *ICSI* intracytoplasmic sperm injection, *NA* not applicable

Continuous variables were displayed as the median along with the 25th and 75th percentiles, and their comparisons were conducted using the Mann-Whitney U test. Categorical variables were presented as the number and percentage, and their comparisons were performed using the Chi-square test

*P* values less than 0.050 were shown in bold

### Impacts of FCPs on the whole population undergoing IVF/ICSI treatment

Our results showed that there were no significant differences in embryological outcomes (oocytes retrieved, normal fertilization rate, cleavage rate, and transplantable embryo rate) (Table S1) and clinical outcomes (biochemical pregnancy rate, clinical pregnancy rate, miscarriage rate, preterm birth rate, and live birth rate) (Table S2) between the FCP and control group. Regarding the subgroups, none of them exhibited worse IVF/ICSI outcomes (Table S3, Table 2, and Table 3). The live birth rate of the qh + subgroup was even higher than that of the control group, after adjustment for AFC, diminished ovarian reserve, and paternal factor (24.7% vs. 19.1%, aRR(95%CI) = 1.28(1.05 ~ 1.56), adjusted *P* = 0.014, Table 3).

**Table 2:** Comparison of embryological outcomes between the control and FCP subgroups

	EMM ± SEM	Coefficient (95% CI)	Adjusted *P* value
Oocytes retrieved			
Control	12.1 ± 0.1	Ref.	Ref.
qh+	11.5 ± 0.4	−0.59(−1.30 ~ 0.12)	0.103
pstk+	12.4 ± 0.4	0.35(−0.41 ~ 1.11)	0.363
inv(9)	11.9 ± 0.6	−0.19(−1.41 ~ 1.02)	0.754
multiple	13.0 ± 0.7	0.95(− 0.43 ~ 2.33)	0.175
others	11.9 ± 0.7	−0.20(− 1.65 ~ 1.24)	0.783
Normal fertilization rate (%)			
Control	60.6 ± 0.2	Ref.	Ref.
qh+	61.6 ± 1.3	0.01(−0.02 ~ 0.04)	0.466
pstk+	60.6 ± 1.4	0.00(−0.03 ~ 0.03)	0.974
inv(9)	65.0 ± 2.3	0.04(0.00 ~ 0.09)	0.063
multiple	56.6 ± 2.6	−0.04(−0.09 ~ 0.01)	0.124
others	57.8 ± 2.8	−0.03(− 0.08 ~ 0.03)	0.298
Cleavage rate (%)			
Control	97.9 ± 0.1	Ref.	Ref.
qh+	98.2 ± 0.5	0.00(−0.01 ~ 0.01)	0.487
pstk+	97.3 ± 0.5	−0.01(− 0.02 ~ 0.00)	0.258
inv(9)	97.5 ± 0.8	0.00(−0.02 ~ 0.01)	0.595
multiple	98.2 ± 0.9	0.00(−0.02 ~ 0.02)	0.773
others	96.8 ± 1.0	−0.01(− 0.03 ~ 0.01)	0.260
Transplantable embryo rate (%)			
Control	54.3 ± 0.3	Ref.	Ref.
qh+	54.2 ± 1.7	0.00(−0.03 ~ 0.03)	0.958
pstk+	52.0 ± 1.8	−0.02(− 0.06 ~ 0.01)	0.205
inv(9)	58.1 ± 2.8	0.04(−0.02 ~ 0.09)	0.183
multiple	49.4 ± 3.2	−0.05(− 0.11 ~ 0.01)	0.128
others	58.5 ± 3.4	0.04(−0.03 ~ 0.11)	0.222

*FCP* female chromosomal polymorphism, *EMM* estimated marginal mean, *SEM* standard error of the means, *CI* confidence interval, *Ref* reference

The EMMs, coefficients with their corresponding 95% CIs, and adjusted P values were calculated using generalized linear regression models. The embryological outcomes were adjusted for AFC, diminished ovarian reserve, and paternal factor, except that the oocytes retrieved were adjusted only for AFC and diminished ovarian reserve

**Table 3:** Comparison of clinical outcomes between the control and FCP subgroups^a^

	Rate (cases/study subjects (%))	aRR (95% CI)	Adjusted *P* value
Biochemical pregnancy			
Control	3461/9298(37.2)	Ref.	Ref.
qh+	125/312(40.1)	1.06(0.93 ~ 1.22)	0.389
pstk+	98/269(36.4)	0.97(0.83 ~ 1.14)	0.707
inv(9)	39/100(39.0)	1.02(0.79 ~ 1.30)	0.903
multiple	34/78(43.6)	1.17(0.91 ~ 1.51)	0.212
others	33/74(44.6)	1.17(0.91 ~ 1.50)	0.219
Clinical pregnancy			
Control	2861/9298(30.8)	Ref.	Ref.
qh+	104/312(33.3)	1.07(0.91 ~ 1.26)	0.402
pstk+	83/269(30.9)	1.00(0.83 ~ 1.19)	0.957
inv(9)	33/100(33.0)	1.04(0.79 ~ 1.38)	0.789
multiple	26/78(33.3)	1.08(0.79 ~ 1.48)	0.614
others	29/74(39.2)	1.26(0.95 ~ 1.67)	0.113
Miscarriage			
Control	951/2861(33.2)	Ref.	Ref.
qh+	25/104(24.0)	0.73(0.52 ~ 1.04)	0.079
pstk+	28/83(33.7)	1.02(0.75 ~ 1.39)	0.890
inv(9)	12/33(36.4)	1.11(0.71 ~ 1.75)	0.650
multiple	8/26(30.8)	0.93(0.52 ~ 1.66)	0.808
others	7/29(24.1)	0.72(0.38 ~ 1.38)	0.325
Preterm birth			
Control	291/1772(16.4)	Ref.	Ref.
qh+	12/77(15.6)	0.94(0.55 ~ 1.60)	0.824
pstk+	10/50(20.0)	1.21(0.69 ~ 2.13)	0.512
inv(9)	1/20(5.0)	0.30(0.04 ~ 2.01)	0.212
multiple	5/16(31.3)	1.85(0.89 ~ 3.88)	0.101
others	2/19(10.5)	0.65(0.18 ~ 2.43)	0.524
Live birth			
Control	1767/9268(19.1)	Ref.	Ref.
qh+	77/312(24.7)	1.28(1.05 ~ 1.56)	**0.014**
pstk+	49/269(18.2)	0.95(0.73 ~ 1.22)	0.672
inv(9)	20/100(20.0)	1.02(0.69 ~ 1.51)	0.919
multiple	16/77(20.8)	1.10(0.71 ~ 1.70)	0.663
others	19/73(26.0)	1.37(0.93 ~ 2.01)	0.115

^a^The number of couples at each stage was depicted in Fig. 1, while the methods employed for calculation were elucidated in the Materials and Methods Section. Couples who were lost to follow-up were excluded from the calculation of the live birth rate

*FCP* female chromosomal polymorphism, *aRR* adjusted risk ratio, *CI* confidence interval, *Ref* reference

Log-binomial regression models with adjustment for AFC, diminished ovarian reserve, and paternal factor were employed to calculate the aRRs with their corresponding 95% CIs and adjusted *P* values

*P* values less than 0.050 were shown in bold

### Impacts of FCPs on the population undergoing IVF

Generally, couples with poor semen quality or experiencing fertilization failure are more prone to receive ICSI treatment, thereby resulting in significant heterogeneity between couples undergoing IVF treatment and those undergoing ICSI treatment. Indeed, an examination of 10,788 control couples unveiled significant disparities in baseline characteristics, such as age, basal FSH, AFC, infertility type, infertility factors, stimulation protocol, and stage of the transferred embryo, between those undergoing IVF treatment (*n* = 7407) and those undergoing ICSI treatment (*n* = 3381) (Table S4). Therefore, we next investigated the impacts of FCPs on the outcomes of IVF and ICSI, respectively.

Fig. S2 displayed the distribution of couples at various stages of IVF treatment. In couples undergoing IVF, the FCP group (*n* = 647) and the control group (n = 7407) differed significantly in the proportions of couples with diminished ovarian reserve and male infertility factors (Table S5), which would be adjusted in subsequent multivariate analyses. When the FCPs were analyzed as a whole, the embryological and clinical outcomes of the FCP group were not significantly different from the control group, except that the FCP group exhibited a higher live birth rate (22.7% vs. 19.0%, aRR(95%CI) = 1.18(1.01 ~ 1.39), adjusted *P* = 0.040) (Table S6 and Table S7). Further comprehensive examinations on the effects of various subgroups of FCPs revealed significantly increased normal fertilization rate (63.0% vs. 59.2%, coefficient (95%CI) = 0.04 (0.01 ~ 0.07), adjusted *P* = 0.022), clinical pregnancy rate (37.0% vs. 30.7%, aRR(95%CI) = 1.20(1.00 ~ 1.44), adjusted *P* = 0.048), and live birth rate (27.0% vs.19.0%, aRR(95%CI) = 1.42(1.13 ~ 1.78), adjusted *P* = 0.003) in the qh + subgroup compared to the control group (Table S8, Table 4, and Table 5). However, the presence of multiple polymorphisms in women (the “multiple” subgroup) increased the risk of preterm birth (62.5% vs. 16.9%, aRR(95%CI) = 3.71(2.14 ~ 6.43), adjusted *P* < 0.001, Table 5). The IVF outcomes of the pstk+ subgroup, inv(9) subgroup, and ‘others’ subgroup were not significantly different from those of the control group (Table 4 and Table 5). These results indicated that various types of FCPs affected IVF outcomes differently.

**Table 4:** Comparison of embryological outcomes between the control and FCP subgroups in couples undergoing IVF

	EMM ± SEM	Coefficient (95% CI)	Adjusted *P* value
Oocytes retrieved			
Control	11.7 ± 0.1	Ref.	Ref.
qh+	10.9 ± 0.5	− 0.76(−1.73 ~ 0.22)	0.128
pstk+	12.2 ± 0.5	0.48(−0.54 ~ 1.50)	0.357
inv(9)	12.2 ± 0.9	0.49(−1.22 ~ 2.19)	0.578
multiple	12.6 ± 1.0	0.90(−0.99 ~ 2.79)	0.352
others	10.8 ± 1.0	−0.88(−2.78 ~ 1.03)	0.368
Normal fertilization rate (%)			
Control	59.2 ± 0.3	Ref.	Ref.
qh+	63.0 ± 1.6	0.04(0.01 ~ 0.07)	**0.022**
pstk+	59.6 ± 1.7	0.00(−0.03 ~ 0.04)	0.819
inv(9)	64.2 ± 2.9	0.05(−0.01 ~ 0.11)	0.085
multiple	56.4 ± 3.2	−0.03(− 0.09 ~ 0.04)	0.386
others	57.7 ± 3.2	−0.02(− 0.08 ~ 0.05)	0.639
Cleavage rate (%)			
Control	97.6 ± 0.1	Ref.	Ref.
qh+	98.4 ± 0.6	0.01(0.00 ~ 0.02)	0.202
pstk+	96.9 ± 0.6	−0.01(−0.02 ~ 0.01)	0.259
inv(9)	96.6 ± 1.1	−0.01(− 0.03 ~ 0.01)	0.358
multiple	97.4 ± 1.2	0.00(−0.03 ~ 0.02)	0.863
others	95.6 ± 1.2	−0.02(− 0.04 ~ 0.00)	0.099
Transplantable embryo rate (%)			
Control	52.4 ± 0.4	Ref.	Ref.
qh+	52.2 ± 1.9	0.00(−0.04 ~ 0.04)	0.922
pstk+	50.9 ± 2.1	−0.02(− 0.06 ~ 0.03)	0.470
inv(9)	54.7 ± 3.4	0.02(−0.05 ~ 0.09)	0.503
multiple	47.2 ± 3.8	−0.05(− 0.13 ~ 0.02)	0.175
others	53.8 ± 3.8	0.01(−0.06 ~ 0.09)	0.713

*FCP* female chromosomal polymorphism, *EMM* estimated marginal mean, *SEM* standard error of the means, *CI* confidence interval, *Ref* reference

The EMMs, coefficients with their corresponding 95% CIs, and adjusted P values were calculated using generalized linear regression models. The embryological outcomes, except for the oocytes retrieved, were adjusted for the paternal factor

*P* values less than 0.050 were shown in bold

**Table 5:** Comparison of clinical outcomes between the control and FCP subgroups in couples undergoing IVF^a^

	Rate (cases/study subjects (%))	aRR (95% CI)	Adjusted *P* value
Biochemical pregnancy			
Control	2381/6379(37.3)	Ref.	Ref.
qh+	91/211(43.1)	1.15(0.98 ~ 1.34)	0.088
pstk+	69/189(36.5)	0.98(0.81 ~ 1.18)	0.802
inv(9)	26/62(41.9)	1.13(0.84 ~ 1.52)	0.412
multiple	22/53(41.5)	1.10(0.80 ~ 1.52)	0.548
others	23/54(42.6)	1.14(0.83 ~ 1.55)	0.426
Clinical pregnancy			
Control	1960/6379(30.7)	Ref.	Ref.
qh+	78/211(37.0)	1.20(1.00 ~ 1.44)	**0.048**
pstk+	58/189(30.7)	1.00(0.80 ~ 1.24)	0.992
inv(9)	24/62(38.7)	1.27(0.92 ~ 1.74)	0.143
multiple	16/53(30.2)	0.98(0.65 ~ 1.48)	0.914
others	22/54(40.7)	1.32(0.96 ~ 1.83)	0.090
Miscarriage			
Control	644/1960(32.9)	Ref.	Ref.
qh+	21/78(26.9)	0.82(0.57 ~ 1.19)	0.298
pstk+	17/58(29.3)	0.89(0.60 ~ 1.34)	0.586
inv(9)	9/24(37.5)	1.14(0.68 ~ 1.92)	0.626
multiple	7/16(43.8)	1.34(0.76 ~ 2.34)	0.312
others	7/22(31.8)	0.97(0.52 ~ 1.79)	0.916
Preterm birth			
Control	205/1212(16.9)	Ref.	Ref.
qh+	9/57(15.8)	0.94(0.51 ~ 1.74)	0.848
pstk+	6/39(15.4)	0.92(0.44 ~ 1.94)	0.821
inv(9)	1/14(7.1)	0.42(0.06 ~ 2.78)	0.368
multiple	5/8(62.5)	3.71(2.14 ~ 6.43)	< **0.001**
others	2/12(16.7)	0.98(0.28 ~ 3.49)	0.975
Live birth			
Control	1209/6362(19.0)	Ref.	Ref.
qh+	57/211(27.0)	1.42(1.13 ~ 1.78)	**0.003**
pstk+	38/189(20.1)	1.06(0.79 ~ 1.41)	0.704
inv(9)	14/62(22.6)	1.19(0.75 ~ 1.90)	0.458
multiple	8/53(15.1)	0.79(0.42 ~ 1.50)	0.467
others	12/53(22.6)	1.19(0.72 ~ 1.96)	0.494

^a^The number of couples at each stage was depicted in Fig. S2, while the methods employed for calculation were elucidated in the Materials and Methods Section. Couples who were lost to follow-up were excluded from the calculation of the live birth rate

*FCP* female chromosomal polymorphism, *aRR* adjusted risk ratio, *CI* confidence interval, *Ref* reference

Log-binomial regression models with adjustment for paternal factor were employed to calculate the aRRs with their corresponding 95% CIs and adjusted P values

*P* values less than 0.050 were shown in bold

### Impacts of FCPs on the population undergoing ICSI

Fig. S3 displayed the distribution of couples at various stages of ICSI treatment. In couples undergoing ICSI, the baseline characteristics were comparable between the FCP group (*n* = 304) and the control group (*n* = 3381) (Table S9). Notably, the FCP group as a whole showed a significantly lower oocyte maturation rate (76.0% vs. 78.8%, coefficient (95%CI) = − 0.03(− 0.05 ~ − 0.01), *P* = 0.008, Table S10), while it was not significantly different from the control group in other embryological outcomes and clinical outcomes (Table S10 and Table S11). Further analyses on different subgroups of FCPs revealed that the oocyte maturation rate in the ‘others’ subgroup was significantly lower than that in the control group (69.0 vs. 78.8%, coefficient (95%CI) = − 0.10(− 0.17 ~ − 0.03), *P* = 0.008, Table S12 and Table 6). A similar trend was also observed in the qh + subgroup, pstk+ subgroup, inv(9) subgroup, and ‘multiple’ subgroup, albeit not reaching statistical significance (Table 6). These results suggested that FCPs, particularly those less prevalent, exert detrimental effects on oocyte maturation. In addition, the preterm birth rate of the pstk+ subgroup was significantly higher than that of the control group (36.4% vs. 15.4%, aRR = 2.37 (1.06 ~ 5.30), *P* = 0.036, Table 7). Interestingly, in contrast to the results observed in couples undergoing IVF, the qh + subgroup in couples undergoing ICSI exhibited a lower normal fertilization rate (58.8% vs. 63.8%, coefficient (95%CI) = − 0.05(− 0.10 ~ 0.00), *P* = 0.032, Table 6), a comparable clinical pregnancy rate (25.7% vs. 30.9%, RR = 0.83(0.60 ~ 1.17), *P* = 0.289, Table 7), and a comparable live birth rate (19.8% vs. 19.2%, RR = 1.03(0.69 ~ 1.54), *P* = 0.880, Table 7), compared with the control group (Table 7). These results suggested that the same FCP could affect the outcomes of IVF and ICSI very differently and qh + in females compromised the effectiveness of ICSI treatment rather than IVF treatment.

**Table 6:** Comparison of embryological outcomes between the control and FCP subgroups in couples undergoing ICSI

	EMM ± SEM	Coefficient (95% CI)	*P* value
Oocytes retrieved			
Control	12.8 ± 0.1	Ref.	Ref.
qh+	12.7 ± 0.7	−0.15(−1.52 ~ 1.21)	0.828
pstk+	13.2 ± 0.8	0.38(− 1.15 ~ 1.90)	0.629
inv(9)	14.0 ± 1.1	1.11(−1.11 ~ 3.33)	0.325
multiple	13.0 ± 1.4	0.16(−2.49 ~ 2.81)	0.906
others	13.9 ± 1.5	1.07(−1.95 ~ 4.10)	0.487
Metaphase II (M II) oocytes			
Control	9.9 ± 0.1	Ref.	Ref.
qh+	9.5 ± 0.6	−0.43(−1.53 ~ 0.66)	0.436
pstk+	10.1 ± 0.6	0.19(−1.04 ~ 1.41)	0.767
inv(9)	10.7 ± 0.9	0.79(−0.99 ~ 2.56)	0.385
multiple	9.4 ± 1.1	−0.53(−2.66 ~ 1.59)	0.622
others	10.0 ± 1.2	0.02(−2.40 ~ 2.44)	0.986
Oocyte maturation rate (%)			
Control	78.8 ± 0.3	Ref.	Ref.
qh+	75.8 ± 1.6	−0.03(−0.06 ~ 0.00)	0.073
pstk+	78.2 ± 1.9	−0.01(− 0.04 ~ 0.03)	0.740
inv(9)	76.5 ± 2.7	−0.02(− 0.08 ~ 0.03)	0.397
multiple	74.6 ± 3.2	−0.04(− 0.11 ~ 0.02)	0.197
others	69.0 ± 3.7	−0.10(− 0.17 ~ − 0.03)	**0.008**
Normal fertilization rate (%)			
Control	63.8 ± 0.4	Ref.	Ref.
qh+	58.8 ± 2.3	−0.05(−0.10 ~ 0.00)	**0.032**
pstk+	62.9 ± 2.6	−0.01(− 0.06 ~ 0.04)	0.722
inv(9)	67.2 ± 3.8	0.03(−0.04 ~ 0.11)	0.367
multiple	56.3 ± 4.5	−0.08(− 0.17 ~ 0.01)	0.099
others	57.8 ± 5.2	−0.06(− 0.16 ~ 0.04)	0.253
Cleavage rate (%)			
Control	98.5 ± 0.1	Ref.	Ref.
qh+	97.8 ± 0.7	−0.01(−0.02 ~ 0.01)	0.341
pstk+	98.3 ± 0.8	0.00(−0.02 ~ 0.01)	0.791
inv(9)	99.1 ± 1.2	0.01(−0.02 ~ 0.03)	0.631
multiple	99.6 ± 1.4	0.01(−0.02 ~ 0.04)	0.439
others	100.0 ± 1.7	0.02(−0.02 ~ 0.05)	0.359
Transplantable embryo rate (%)			
Control	58.5 ± 0.6	Ref.	Ref.
qh+	58.2 ± 3.0	0.00(−0.06 ~ 0.06)	0.914
pstk+	54.5 ± 3.4	−0.04(− 0.11 ~ 0.03)	0.248
inv(9)	65.3 ± 5.0	0.07(−0.03 ~ 0.17)	0.174
multiple	53.0 ± 5.9	−0.06(− 0.17 ~ 0.06)	0.344
others	71.2 ± 7.0	0.13(−0.01 ~ 0.26)	0.071

*FCP* female chromosomal polymorphism, *EMM* estimated marginal mean, *SEM* standard error of the means, *CI* confidence interval, *Ref* reference

The EMMs, coefficients with their corresponding 95% CIs, and P values were calculated using generalized linear regression models

*P* values less than 0.050 were shown in bold

**Table 7:** Comparison of clinical outcomes between the control and FCP subgroups in couples undergoing ICSI^a^

	Rate (cases/study subjects (%))	RR (95% CI)	*P* value
Biochemical pregnancy			
Control	1080/2919(37.0)	Ref.	Ref.
qh+	34/101(33.7)	0.91(0.69 ~ 1.20)	0.505
pstk+	29/80(36.3)	0.98(0.73 ~ 1.32)	0.892
inv(9)	13/38(34.2)	0.93(0.59 ~ 1.44)	0.729
multiple	12/25(48.0)	1.30(0.86 ~ 1.96)	0.214
others	10/20(50.0)	1.35(0.87 ~ 2.10)	0.181
Clinical pregnancy			
Control	901/2919(30.9)	Ref.	Ref.
qh+	26/101(25.7)	0.83(0.60 ~ 1.17)	0.289
pstk+	25/80(31.3)	1.01(0.73 ~ 1.41)	0.941
inv(9)	9/38(23.7)	0.77(0.43 ~ 1.36)	0.365
multiple	10/25(40.0)	1.30(0.80 ~ 2.10)	0.293
others	7/20(35.0)	1.13(0.62 ~ 2.07)	0.681
Miscarriage			
Control	307/901(34.1)	Ref.	Ref.
qh+	4/26(15.4)	0.45(0.18 ~ 1.12)	0.085
pstk+	11/25(44.0)	1.29(0.82 ~ 2.03)	0.267
inv(9)	3/9(33.3)	0.98(0.39 ~ 2.48)	0.963
multiple	1/10(10.0)	0.29(0.05 ~ 1.89)	0.197
others	0/7(0.0)	/	0.999
Preterm birth			
Control	86/560(15.4)	Ref.	Ref.
qh+	3/20(15.0)	0.98(0.34 ~ 2.82)	0.965
pstk+	4/11(36.4)	2.37(1.06 ~ 5.30)	**0.036**
inv(9)	0/6(0.0)	NA	NA
multiple	0/8(0.0)	NA	NA
others	0/7(0.0)	NA	NA
Live birth			
Control	558/2906(19.2)	Ref.	Ref.
qh+	20/101(19.8)	1.03(0.69 ~ 1.54)	0.880
pstk+	11/80(13.8)	0.72(0.41 ~ 1.25)	0.237
inv(9)	6/38(15.8)	0.82(0.39 ~ 1.72)	0.603
multiple	8/24(33.3)	1.74(0.98 ~ 3.07)	0.058
others	7/20(35.0)	1.82(1.00 ~ 3.33)	0.051

^a^The number of couples at each stage was depicted in Fig. S3, while the methods employed for calculation were elucidated in the Materials and Methods Section. Couples who were lost to follow-up were excluded from the calculation of the live birth rate

*FCP* female chromosomal polymorphism, *aRR* adjusted risk ratio, *CI* confidence interval, *Ref* reference, *NA* not applicable

Log-binomial regression models were employed to calculate the aRRs with their corresponding 95% CIs and P values

*P* values less than 0.050 were shown in bold

## Discussion

Our study investigated the influence of FCPs on assisted reproductive outcomes in detail. No significant differences were observed when examining the impact of the overall FCPs on the whole assisted reproduction population. This finding may explain the absence of a relationship between FCPs and assisted reproduction in some previous studies. However, when investigating the effects of various types of FCPs in couples receiving IVF and ICSI treatment respectively, some intriguing results were obtained.

A remarkable finding of the present study is that FCPs impair the maturation of oocytes. Constitutive heterochromatin is known to participate in the silencing of gene expression, maintenance of genome stability, and correct chromosome segregation [20–22]. Polymorphisms or anomalies in these regions were reported to be related to chromosome segregation errors and chromosome aneuploidies [23, 24]. A previous study showed that women with chromosomal polymorphisms have an increased number of aneuploid blastocysts [6]. Another study suggested a correlation between FCPs and the occurrence of multinucleated embryos [25]. Taken together, these findings suggest the hypothesis that FCPs may increase the probability of meiotic errors, leading to a higher prevalence of chromosomal aneuploidy in oocytes and impairing the maturation of oocytes and the subsequent development of embryos. However, this intriguing hypothesis requires further experimental verification.

Our findings suggested an increased vulnerability to preterm birth in women undergoing IVF with multiple polymorphisms and in women undergoing ICSI with pstk+. Another study, which did not distinguish between different types of polymorphisms, also indicated a link between FCPs and a higher preterm birth rate [2]. How FCPs contribute to the increased risk of preterm birth remains uncertain, and whether it is related to the detrimental effects of FCPs on oocyte development, warrants further research.

An unexpected finding was the significantly distinct effects of qh + on the outcomes of IVF and ICSI. Our findings demonstrated that women who carried qh + and underwent IVF treatment exhibited higher rates of fertilization, clinical pregnancy, and live birth. A previous study indicated that women with chromosomal polymorphisms had a higher clinical pregnancy rate and live birth rate [16]. However, this study did not specifically examine the influence of different types of polymorphisms, nor did it separately analyze the impact of polymorphisms on the outcomes of IVF and ICSI. In contrast to the observations in couples undergoing IVF, the present study showed that qh + in females had a detrimental effect on ICSI outcomes, notably reducing the fertilization rate. Hence, for couples who are recommended for ICSI treatment due to poor semen quality in males or previous fertilization failures, the expected effects may not be achieved if the female partners carry qh + .

Intriguingly, our recent publication [26] on MCPs showed that couples who underwent ICSI and carried pstk+ in males also exhibited a higher risk of preterm birth, suggesting that MCPs and FCPs share similar influences in the risk of preterm birth. However, the rare polymorphisms have a significant impact on gamete quality in females, while in males, it is inv(9) and 9qh + that significantly affect gamete quality. In addition, male Yqh + is related to an increased risk of preterm birth in couples undergoing ICSI, suggesting that Y chromosome polymorphisms in males bring additional risks for couples undergoing ICSI.

### Strengths and limitations

One of the strengths of the present study is that we excluded MCPs and focused on the impact of FCPs. Additionally, the large sample size enables us to explore the associations of different types of FCPs with the outcomes of IVF and ICSI, respectively. However, several limitations should be noted. First, despite our efforts to analyze as many baseline characteristics as possible and adjust for potential confounders, the results may be affected by unidentified confounders. Second, our investigation is a single-center study conducted in the northern Chinese population, limiting the generalizability of the findings to other populations. Third, retrospective study may be subject to recall bias and reverse causation bias, and cannot elucidate the underlying biological mechanisms of the findings. Additionally, the relatively small sample sizes in some subgroups reduce the power of statistical analysis. Therefore, further investigation through larger-scale prospective cohort studies or even randomized controlled trials, coupled with in-depth mechanistic studies, will be beneficial in elucidating the impact of FCPs on assisted reproductive outcomes.

## Conclusions

Our study revealed remarkable heterogeneity in the impacts of various FCPs on IVF and ICSI outcomes, and these findings will contribute to developing personalized treatment strategies according to the type of FCPs and the fertilization method. In the future, larger prospective cohort studies, randomized controlled trials, and functional studies are warranted to comprehensively elucidate the impact of each type of FCPs on assisted reproductive health.

### Supplementary Information

**Supplementary Material 1.**

## Data Availability

The datasets used and/or analyzed during the current study are available from the corresponding author on reasonable request.
