# Supplementary material for: Diverse impacts of female chromosomal polymorphisms on assisted reproduction outcomes: a retrospective cohort study
Source: BMC Pregnancy Childbirth. 2024 Apr 27;24:331. doi: 10.1186/s12884-024-06532-w (PMC11055351; doi:10.1186/s12884-024-06532-w)
Supplement: Supplementary file 1 — Supplementary Material 1. [file 12884_2024_6532_MOESM1_ESM.docx]

**Supplementary Materials for**

**Diverse impacts of female chromosomal polymorphisms on assisted reproduction outcomes: a retrospective cohort study**

**Authors:**

Yongjie Lu^1,2,3,4,5^, Tian Tian^1,2,3,4,5^, Lixue Chen^1,2,3,4,5^, Liying Yan^1,2,3,4,5^, Liang Chang^1,2,3,4,5,^*, Jie Qiao^1,2,3,4,5,^*

^1^Center for Reproductive Medicine, Department of Obstetrics and Gynecology, Peking University Third Hospital, Beijing, 100191, China

^2^National Clinical Research Center for Obstetrics and Gynecology (Peking University Third Hospital), Beijing, 100191, China

^3^Key Laboratory of Assisted Reproduction (Peking University), Ministry of Education, Beijing, 100191, China

^4^Beijing Key Laboratory of Reproductive Endocrinology and Assisted Reproductive Technology, Beijing, 100191, China

^5^State Key Laboratory of Female Fertility Promotion, Center for Reproductive Medicine, Department of Obstetrics and Gynecology, Peking University Third Hospital, Beijing 100191, China.

*Correspondence:

Liang Chang: changliangchina@163.com, Tel: 86-10-82265073;

Jie Qiao: jie.qiao@263.net, Tel: 86-10-82265080.

**Table of contents**

Supplementary Tables

Table S1 Comparison of embryological outcomes between the control and FCP group

Table S2 Comparison of clinical outcomes between the control and FCP group

Table S3 Comparison of baseline characteristics between the control and FCP subgroups

Table S4 Comparison of baseline characteristics between couples undergoing IVF and ICSI in the control group

Table S5 Comparison of baseline characteristics between the control and FCP group in couples undergoing IVF

Table S6 Comparison of embryological outcomes between the control and FCP group in couples undergoing IVF

Table S7 Comparison of clinical outcomes between the control and FCP group in couples undergoing IVF

Table S8 Comparison of baseline characteristics between the control and FCP subgroups in couples undergoing IVF

Table S9 Comparison of baseline characteristics between the control and FCP group in couples undergoing ICSI

Table S10 Comparison of embryological outcomes between the control and FCP group in couples undergoing ICSI

Table S11 Comparison of clinical outcomes between the control and FCP group in couples undergoing ICSI

Table S12 Comparison of baseline characteristics between the control and FCP subgroups in couples undergoing ICSI

Figures

Fig. S1 Representative images of chromosomal polymorphisms

Fig. S2 Flow chart of participants at each stage of IVF treatment

Fig. S3 Flow chart of participants at each stage of ICSI treatment

| **Table S1 Comparison of embryological outcomes between the control and FCP group** | | | | |
| --- | --- | --- | --- | --- |
|  | **EMM±SEM** | | **Coefficient (95% CI)** | adjusted P value |
|  | **Control n=10,788** | **FCP group n=951** |  |  |
| **Oocytes retrieved** | 12.1±0.1 | 11.9±0.2 | -0.12(-0.59~0.35) | 0.612 |
| **Normal fertilization rate (%)** | 60.6±0.2 | 60.9±0.8 | 0.00(-0.01~0.02) | 0.784 |
| **Cleavage rate(%)** | 97.9±0.1 | 97.7±0.3 | 0.00(-0.01~0.00) | 0.523 |
| **Transplantable embryo rate (%)** | 54.3±0.3 | 53.9±1.0 | 0.00(-0.03~0.02) | 0.681 |
| FCP, female chromosomal polymorphism; EMM, estimated marginal mean; SEM, standard error of the means; CI, confidence interval | | | | |
| The EMMs, coefficients with their corresponding 95% CIs, and adjusted P values were calculated using generalized linear regression models. The embryological outcomes were adjusted for diminished ovarian reserve and paternal factor, except that the oocytes retrieved were adjusted only for diminished ovarian reserve. | | | | |

| **Table S2 Comparison of clinical outcomes between the control and FCP group^a^** | | | | |
| --- | --- | --- | --- | --- |
|  | **Control** | **FCP group** | **aRR (95% CI)** | **adjusted *P* value** |
| **Biochemical pregnancy rate** (cases/study subjects (%)) | 3,461/9,298(37.2) | 329/833(39.5) | 1.05(0.96~1.15) | 0.269 |
| **Clinical pregnancy rate** (cases/study subjects (%)) | 2,861/9,298(30.8) | 275/833(33.0) | 1.06(0.96~1.18) | 0.243 |
| **Miscarriage rate** (cases/study subjects (%)) | 951/2,861(33.2) | 80/275(29.1) | 0.88(0.73~1.07) | 0.205 |
| **Preterm birth rate** (cases/study subjects (%)) | 291/1,772(16.4) | 30/182(16.5) | 1.00(0.71~1.40) | 0.975 |
| **Live birth rate** (cases/study subjects (%)) | 1,767/9,268(19.1) | 181/831(21.8) | 1.13(0.99~1.30) | 0.073 |
| ^a^The number of couples at each stage was depicted in Fig. 1, while the methods employed for calculation were elucidated in the Materials and Methods Section. Couples who were lost to follow-up were excluded from the calculation of the live birth rate. | | | | |
| FCP, female chromosomal polymorphism; aRR, adjusted risk ratio; CI, confidence interval. | | | | |
| Log-binomial regression models with adjustment for diminished ovarian reserve and paternal factor were employed to calculate the aRRs with their corresponding 95% CIs and adjusted P values. | | | | |

| **Table S3 Comparison of baseline characteristics between the control and FCP subgroups** | | | | | | | |
| --- | --- | --- | --- | --- | --- | --- | --- |
|  | **Control n=10,788** | **qh+ n=352** | **pstk+ n=307** | **inv(9) n=118** | **multiple n=91** | **others n=83** | ***P* value** |
| **Maternal age, years** | 32.0(30.0~35.0) | 32.0(30.0~35.0) | 32.0(30.0~35.0) | 33.0(30.0~36.0) | 32.0(30.0~35.0) | 33.0(30.0~35.0) | 0.719 |
| **Paternal age, years** | 33.0(30.0~36.0) | 33.0(30.0~36.0) | 33.0(30.0~36.0) | 34.0(30.0~37.0) | 33.0(30.0~36.0) | 32.0(30.0~36.0) | 0.668 |
| **Maternal BMI, kg/m2** | 22.2(20.2~24.8) | 22.0(20.3~25.1) | 22.0(20.0~24.8) | 22.7(20.6~25.4) | 22.0(19.9~24.3) | 22.0(20.4~25.1) | 0.668 |
| **Paternal BMI, kg/m2** | 25.1(23.0~27.7) | 24.7(22.9~27.8) | 25.7(23.6~28.0) | 24.6(22.8~26.9) | 24.2(23.1~27.0) | 25.9(22.5~28.0) | 0.275 |
| **Basal FSH level** | 6.3(4.8~7.9) | 6.1(4.7~7.8) | 6.2(4.8~7.8) | 6.4(5.2~7.8) | 6.7(5.5~8.8) | 6.3(4.5~7.6) | 0.304 |
| **Basal E2 level** | 155.0(114.0~200.0) | 153.0(110.0~199.0) | 149.0(110.0~198.5) | 157.0(123.5~193.0) | 152.0(118.0~194.9) | 166.0(118.6~219.5) | 0.798 |
| **AFC** | 11.0(8.0~15.0) | 10.5(8.0~15.0) | 10.0(8.0~15.0) | 13.0(9.0~17.0) | 10.0(7.0~14.0) | 10.0(6.0~14.0) | **0.014** |
| **Infertility type** |  |  |  |  |  |  | 0.293 |
| Primary | 6,354(58.9) | 198(56.3) | 189(61.6) | 74(62.7) | 45(49.5) | 47(56.6) |  |
| Secondary | 4,434(41.1) | 154(43.8) | 118(38.4) | 44(37.3) | 46(50.5) | 36(43.4) |  |
| **Tubal factor** |  |  |  |  |  |  | 0.838 |
| No | 6,770(62.8) | 214(60.8) | 197(64.2) | 74(62.7) | 54(59.3) | 56(67.5) |  |
| Yes | 4,018(37.2) | 138(39.2) | 110(35.8) | 44(37.3) | 37(40.7) | 27(32.5) |  |
| **PCOS** |  |  |  |  |  |  | 0.620 |
| No | 8,878(82.3) | 299(84.9) | 252(82.1) | 96(81.4) | 75(82.4) | 73(88.0) |  |
| Yes | 1,910(17.7) | 53(15.1) | 55(17.9) | 22(18.6) | 16(17.6) | 10(12.0) |  |
| **Diminished ovarian reserve** |  |  |  |  |  |  | **0.043** |
| No | 9,345(86.6) | 315(89.5) | 273(88.9) | 112(94.9) | 76(83.5) | 73(88.0) |  |
| Yes | 1,443(13.4) | 37(10.5) | 34(11.1) | 6(5.1) | 15(16.5) | 10(12.0) |  |
| **Endometriosis** |  |  |  |  |  |  | 0.550 |
| No | 9,686(89.8) | 318(90.3) | 274(89.3) | 110(93.2) | 84(92.3) | 71(85.5) |  |
| Yes | 1,102(10.2) | 34(9.7) | 33(10.7) | 8(6.8) | 7(7.7) | 12(14.5) |  |
| **Other maternal factors** |  |  |  |  |  |  | 0.456 |
| No | 8,711(80.7) | 288(81.8) | 240(78.2) | 96(81.4) | 80(87.9) | 66(79.5) |  |
| Yes | 2,077(19.3) | 64(18.2) | 67(21.8) | 22(18.6) | 11(12.1) | 17(20.5) |  |
| **Paternal factor** |  |  |  |  |  |  | **0.014** |
| No | 5,167(47.9) | 190(54.0) | 166(54.1) | 51(43.2) | 53(58.2) | 42(50.6) |  |
| Yes | 5,621(52.1) | 162(46.0) | 141(45.9) | 67(56.8) | 38(41.8) | 41(49.4) |  |
| **Stimulation protocol** |  |  |  |  |  |  | 0.304 |
| GnRH agonist | 3,689(34.2) | 124(35.2) | 112(36.5) | 34(28.8) | 39(42.9) | 32(38.6) |  |
| GnRH antagonist | 7,099(65.8) | 228(64.8) | 195(63.5) | 84(71.2) | 52(57.1) | 51(61.4) |  |
| **Fertilization type** |  |  |  |  |  |  | 0.742 |
| IVF | 7,407(68.7) | 236(67.0) | 215(70.0) | 75(63.6) | 61(67.0) | 60(72.3) |  |
| ICSI | 3,381(31.3) | 116(33.0) | 92(30.0) | 43(36.4) | 30(33.0) | 23(27.7) |  |
| **Embryo transfer method** |  |  |  |  |  |  | 0.429 |
| Fresh | 6,769(62.7) | 225(63.9) | 190(61.9) | 73(61.9) | 54(59.3) | 63(75.9) |  |
| Frozen | 2,529(23.4) | 87(24.7) | 79(25.7) | 27(22.9) | 24(26.4) | 11(13.3) |  |
| No transfer | 1,490(13.8) | 40(11.4) | 38(12.4) | 18(15.3) | 13(14.3) | 9(10.8) |  |
| **Stage of transferred embryo** |  |  |  |  |  |  | 0.338 |
| Cleavage stage | 8,041(86.5) | 266(85.3) | 230(85.5) | 87(87.0) | 62(79.5) | 68(91.9) |  |
| Blastocyst stage | 1,257(13.5) | 46(14.7) | 39(14.5) | 13(13.0) | 16(20.5) | 6(8.1) |  |
| **Number of transferred embryo** |  |  |  |  |  |  | 0.842 |
| one | 1,991(21.4) | 64(20.5) | 64(23.8) | 23(23.0) | 20(25.6) | 15(20.3) |  |
| two | 7,307(78.6) | 248(79.5) | 205(76.2) | 77(77.0) | 58(74.4) | 59(79.7) |  |
| FCP, female chromosomal polymorphism; BMI, body mass index; FSH, follicle stimulating hormone; AFC, antral follicle count; PCOS, polycystic ovarian syndrome; IVF, in vitro fertilization; ICSI, intracytoplasmic sperm injection. | | | | | | | |
| Continuous variables were displayed as the median along with the 25th and 75th percentiles, and their comparisons were conducted using the Kruskal–Wallis test. Categorical variables were presented as the number and percentage, and their comparisons were performed using the Chi-square test. | | | | | | | |
| P values less than 0.050 were shown in bold. | | | | | | | |

| **Table S4 Comparison of baseline characteristics between couples undergoing IVF and ICSI in the control group** | | | |
| --- | --- | --- | --- |
|  | **IVF n=7,407** | **ICSI n=3,381** | **P value** |
| **Maternal age, years** | 32.0(30.0~35.0) | 32.0(29.0~34.0) | **< 0.001** |
| **Paternal age, years** | 33.0(30.0~36.0) | 33.0(30.0~36.0) | **< 0.001** |
| **Maternal BMI, kg/m2** | 22.2(20.3~24.8) | 22.0(20.2~24.8) | 0.107 |
| **Paternal BMI, kg/m2** | 25.1(23.0~27.7) | 25.1(22.9~27.8) | 0.562 |
| **Basal FSH level** | 6.4(4.8~8.0) | 6.2(4.8~7.8) | **0.002** |
| **Basal E2 level** | 156.0(114.0~200.0) | 154.0(115.0~199.0) | 0.973 |
| **AFC** | 10.0(7.0~14.0) | 11.0(8.0~15.0) | **< 0.001** |
| **Infertility type** |  |  | **< 0.001** |
| Primary | 3,982(53.8) | 2,372(70.2) |  |
| Secondary | 3,425(46.2) | 1,009(29.8) |  |
| **Tubal factor** |  |  | **< 0.001** |
| No | 4,046(54.6) | 2,724(80.6) |  |
| Yes | 3,361(45.4) | 657(19.4) |  |
| **PCOS** |  |  | **< 0.001** |
| No | 6,026(81.4) | 2,852(84.4) |  |
| Yes | 1,381(18.6) | 529(15.6) |  |
| **Diminished ovarian reserve** |  |  | **< 0.001** |
| No | 6,273(84.7) | 3,072(90.9) |  |
| Yes | 1,134(15.3) | 309(9.1) |  |
| **Endometriosis** |  |  | **< 0.001** |
| No | 6,544(88.3) | 3,142(92.9) |  |
| Yes | 863(11.7) | 239(7.1) |  |
| **Other maternal factors** |  |  | **< 0.001** |
| No | 5,897(79.6) | 2,814(83.2) |  |
| Yes | 1,510(20.4) | 567(16.8) |  |
| **Paternal factor** |  |  | **< 0.001** |
| No | 4,225(57.0) | 942(27.9) |  |
| Yes | 3,182(43.0) | 2,439(72.1) |  |
| **Stimulation protocol** |  |  | **< 0.001** |
| GnRH agonist | 2,455(33.1) | 1,234(36.5) |  |
| GnRH antagonist | 4,952(66.9) | 2,147(63.5) |  |
| **Embryo transfer method** |  |  | 0.881 |
| Fresh | 4,652(62.8) | 2,117(62.6) |  |
| Frozen | 1,727(23.3) | 802(23.7) |  |
| No transfer | 1,028(13.9) | 462(13.7) |  |
| **Stage of transferred embryo** |  |  | **0.007** |
| Cleavage stage | 5,475(85.8) | 2,566(87.9) |  |
| Blastocyst stage | 904(14.2) | 353(12.1) |  |
| **Number of transferred embryo** |  |  | 0.056 |
| one | 1,401(22.0) | 590(20.2) |  |
| two | 4,978(78.0) | 2,329(79.8) |  |
| BMI, body mass index; FSH, follicle stimulating hormone; AFC, antral follicle count; PCOS, polycystic ovarian syndrome; IVF, in vitro fertilization; ICSI, intracytoplasmic sperm injection. | | | |
| Continuous variables were displayed as the median along with the 25th and 75th percentiles, and their comparisons were conducted using the Mann-Whitney U test. Categorical variables were presented as the number and percentage, and their comparisons were performed using the Chi-square test. | | | |
| P values less than 0.050 were shown in bold. | | | |

| **Table S5 Comparison of baseline characteristics between the control and FCP group in couples undergoing IVF** | | | |
| --- | --- | --- | --- |
|  | **Control n=7,407** | **FCP group n=647** | **P value** |
| **Maternal age, years** | 32.0(30.0~35.0) | 33.0(30.0~35.0) | 0.612 |
| **Paternal age, years** | 33.0(30.0~36.0) | 33.0(30.0~36.0) | 0.287 |
| **Maternal BMI, kg/m2** | 22.2(20.3~24.8) | 22.3(20.3~25.0) | 0.678 |
| **Paternal BMI, kg/m2** | 25.1(23.0~27.7) | 25.1(23.1~27.7) | 0.784 |
| **Basal FSH level** | 6.4(4.8~8.0) | 6.3(4.9~8.0) | 0.811 |
| **Basal E2 level** | 156.0(114.0~200.0) | 152.0(110.0~195.0) | 0.365 |
| **AFC** | 10.0(7.0~14.0) | 10.0(8.0~15.0) | 0.552 |
| **Infertility type** |  |  | 0.929 |
| Primary | 3,982(53.8) | 349(53.9) |  |
| Secondary | 3,425(46.2) | 298(46.1) |  |
| **Tubal factor** |  |  | 0.476 |
| No | 4,046(54.6) | 344(53.2) |  |
| Yes | 3,361(45.4) | 303(46.8) |  |
| **PCOS** |  |  | 0.654 |
| No | 6,026(81.4) | 531(82.1) |  |
| Yes | 1,381(18.6) | 116(17.9) |  |
| **Diminished ovarian reserve** |  |  | **0.035** |
| No | 6,273(84.7) | 568(87.8) |  |
| Yes | 1,134(15.3) | 79(12.2) |  |
| **Endometriosis** |  |  | 0.452 |
| No | 6,544(88.3) | 578(89.3) |  |
| Yes | 863(11.7) | 69(10.7) |  |
| **Other maternal factors** |  |  | 0.346 |
| No | 5,897(79.6) | 505(78.1) |  |
| Yes | 1,510(20.4) | 142(21.9) |  |
| **Paternal factor** |  |  | **0.004** |
| No | 4,225(57.0) | 407(62.9) |  |
| Yes | 3,182(43.0) | 240(37.1) |  |
| **Stimulation protocol** |  |  | 0.278 |
| GnRH agonist | 2,455(33.1) | 228(35.2) |  |
| GnRH antagonist | 4,952(66.9) | 419(64.8) |  |
| **Embryo transfer method** |  |  | 0.375 |
| Fresh | 4,652(62.8) | 409(63.2) |  |
| Frozen | 1,727(23.3) | 160(24.7) |  |
| No transfer | 1,028(13.9) | 78(12.1) |  |
| **Stage of transferred embryo** |  |  | 0.538 |
| Cleavage stage | 5,475(85.8) | 483(84.9) |  |
| Blastocyst stage | 904(14.2) | 86(15.1) |  |
| **Number of transferred embryo** |  |  | 0.626 |
| one | 1,401(22.0) | 130(22.8) |  |
| two | 4,978(78.0) | 439(77.2) |  |
| **Type of FCPs** |  |  |  |
| qh+ | NA | 236(36.5) |  |
| pstk+ | NA | 215(33.2) |  |
| inv(9) | NA | 75(11.6) |  |
| multiple | NA | 61(9.4) |  |
| others | NA | 60(9.3) |  |
| FCP, female chromosomal polymorphism; BMI, body mass index; FSH, follicle stimulating hormone; AFC, antral follicle count; PCOS, polycystic ovarian syndrome; IVF, in vitro fertilization; ICSI, intracytoplasmic sperm injection; NA, not applicable. | | | |
| Continuous variables were displayed as the median along with the 25th and 75th percentiles, and their comparisons were conducted using the Mann-Whitney U test. Categorical variables were presented as the number and percentage, and their comparisons were performed using the Chi-square test. | | | |
| P values less than 0.050 were shown in bold. | | | |

| **Table S6 Comparison of embryological outcomes between the control and FCP group in couples undergoing IVF** | | | | |
| --- | --- | --- | --- | --- |
|  | **EMM±SEM** | | **Coefficient (95% CI)** | **adjusted *P* value** |
|  | **Control n=7,407** | **FCP group n=647** |  |  |
| **Oocytes retrieved** | 11.7±0.1 | 11.4±0.3 | -0.28(-0.84~0.29) | 0.341 |
| **Normal fertilization rate (%)** | 59.2±0.3 | 60.8±1.0 | 0.02(0.00~0.04) | 0.116 |
| **Cleavage rate(%)** | 97.6±0.1 | 97.3±0.4 | 0.00(-0.01~0.01) | 0.469 |
| **Transplantable embryo rate (%)** | 52.4±0.4 | 51.7±1.2 | -0.01(-0.03~0.02) | 0.571 |
| FCP, female chromosomal polymorphism; EMM, estimated marginal mean; SEM, standard error of the means; CI, confidence interval | | | | |
| The EMMs, coefficients with their corresponding 95% CIs, and adjusted P values were calculated using generalized linear regression models. The embryological outcomes were adjusted for diminished ovarian reserve and paternal factor, except that the oocytes retrieved were adjusted only for diminished ovarian reserve. | | | | |

| **Table S7 Comparison of clinical outcomes between the control and FCP group in couples undergoing IVF^a^** | | | | |
| --- | --- | --- | --- | --- |
|  | **Control** | **FCP group** | **aRR (95% CI)** | **adjusted *P* value** |
| **Biochemical pregnancy rate** (cases/study subjects (%)) | 2,381/6,379(37.3) | 231/569(40.6) | 1.07(0.97~1.19) | 0.177 |
| **Clinical pregnancy rate** (cases/study subjects (%)) | 1,960/6,379(30.7) | 198/569(34.8) | 1.12(0.99~1.26) | 0.065 |
| **Miscarriage rate** (cases/study subjects (%)) | 644/1,960(32.9) | 61/198(30.8) | 0.94(0.76~1.17) | 0.590 |
| **Preterm birth rate** (cases/study subjects (%)) | 205/1,212(16.9) | 23/130(17.7) | 1.04(0.70~1.54) | 0.841 |
| **Live birth rate** (cases/study subjects (%)) | 1,209/6,362(19.0) | 129/568(22.7) | 1.18(1.01~1.39) | **0.040** |
| ^a^The number of couples at each stage was depicted in Fig. S2, while the methods employed for calculation were elucidated in the Materials and Methods Section. Couples who were lost to follow-up were excluded from the calculation of the live birth rate. | | | | |
| FCP, female chromosomal polymorphism; aRR, adjusted risk ratio; CI, confidence interval. | | | | |
| Log-binomial regression models with adjustment for diminished ovarian reserve and paternal factor were employed to calculate the aRRs with their corresponding 95% CIs and adjusted P values. | | | | |
| P values less than 0.050 were shown in bold. | | | | |

| **Table S8 Comparison of baseline characteristics between the control and FCP subgroups in couples undergoing IVF** | | | | | | | |
| --- | --- | --- | --- | --- | --- | --- | --- |
|  | **Control n=7,407** | **qh+ n=236** | **pstk+ n=215** | **inv(9) n=75** | **multiple n=61** | **others n=60** | ***P* value** |
| **Maternal age, years** | 32.0(30.0~35.0) | 32.0(30.0~35.0) | 32.0(30.0~35.0) | 34.0(30.5~36.0) | 33.0(30.0~35.0) | 33.0(31.0~35.0) | 0.350 |
| **Paternal age, years** | 33.0(30.0~36.0) | 33.0(30.0~36.0) | 33.0(30.0~36.0) | 34.0(31.0~37.0) | 33.0(31.0~36.0) | 33.0(30.0~36.0) | 0.479 |
| **Maternal BMI, kg/m2** | 22.2(20.3~24.8) | 22.3(20.5~25.1) | 22.0(20.1~24.8) | 22.8(20.6~25.4) | 22.6(20.0~24.1) | 22.3(20.2~25.1) | 0.874 |
| **Paternal BMI, kg/m2** | 25.1(23.0~27.7) | 24.7(23.0~27.7) | 25.8(23.6~28.2) | 24.4(22.7~26.1) | 24.2(23.0~26.8) | 25.7(23.2~27.8) | 0.149 |
| **Basal FSH level** | 6.4(4.8~8.0) | 6.1(4.8~7.8) | 6.2(4.6~8.0) | 6.3(5.3~7.8) | 6.4(5.3~8.7) | 6.8(4.6~8.6) | 0.561 |
| **Basal E2 level** | 156.0(114.0~200.0) | 149.0(108.0~193.1) | 152.0(108.0~195.0) | 162.5(133.0~193.0) | 144.0(112.5~187.5) | 164.5(111.0~221.5) | 0.495 |
| **AFC** | 10.0(7.0~14.0) | 10.0(8.0~14.0) | 10.0(7.0~16.0) | 13.0(9.0~16.0) | 10.0(7.0~14.0) | 10.0(6.0~12.0) | 0.059 |
| **Infertility type** |  |  |  |  |  |  | 0.439 |
| Primary | 3,982(53.8) | 121(51.3) | 125(58.1) | 42(56.0) | 27(44.3) | 34(56.7) |  |
| Secondary | 3,425(46.2) | 115(48.7) | 90(41.9) | 33(44.0) | 34(55.7) | 26(43.3) |  |
| **Tubal factor** |  |  |  |  |  |  | 0.550 |
| No | 4,046(54.6) | 119(50.4) | 122(56.7) | 41(54.7) | 28(45.9) | 34(56.7) |  |
| Yes | 3,361(45.4) | 117(49.6) | 93(43.3) | 34(45.3) | 33(54.1) | 26(43.3) |  |
| **PCOS** |  |  |  |  |  |  | 0.709 |
| No | 6,026(81.4) | 197(83.5) | 176(81.9) | 59(78.7) | 47(77.0) | 52(86.7) |  |
| Yes | 1,381(18.6) | 39(16.5) | 39(18.1) | 16(21.3) | 14(23.0) | 8(13.3) |  |
| **Diminished ovarian reserve** |  |  |  |  |  |  | 0.112 |
| No | 6,273(84.7) | 211(89.4) | 185(86.0) | 70(93.3) | 50(82.0) | 52(86.7) |  |
| Yes | 1,134(15.3) | 25(10.6) | 30(14.0) | 5(6.7) | 11(18.0) | 8(13.3) |  |
| **Endometriosis** |  |  |  |  |  |  | 0.370 |
| No | 6,544(88.3) | 211(89.4) | 189(87.9) | 72(96.0) | 55(90.2) | 51(85.0) |  |
| Yes | 863(11.7) | 25(10.6) | 26(12.1) | 3(4.0) | 6(9.8) | 9(15.0) |  |
| **Other maternal factors** |  |  |  |  |  |  | 0.670 |
| No | 5,897(79.6) | 187(79.2) | 164(76.3) | 56(74.7) | 51(83.6) | 47(78.3) |  |
| Yes | 1,510(20.4) | 49(20.8) | 51(23.7) | 19(25.3) | 10(16.4) | 13(21.7) |  |
| **Paternal factor** |  |  |  |  |  |  | **0.001** |
| No | 4,225(57.0) | 158(66.9) | 131(60.9) | 38(50.7) | 46(75.4) | 34(56.7) |  |
| Yes | 3,182(43.0) | 78(33.1) | 84(39.1) | 37(49.3) | 15(24.6) | 26(43.3) |  |
| **Stimulation protocol** |  |  |  |  |  |  | 0.186 |
| GnRH agonist | 2,455(33.1) | 83(35.2) | 77(35.8) | 18(24.0) | 25(41.0) | 25(41.7) |  |
| GnRH antagonist | 4,952(66.9) | 153(64.8) | 138(64.2) | 57(76.0) | 36(59.0) | 35(58.3) |  |
| **Embryo transfer method** |  |  |  |  |  |  | 0.243 |
| Fresh | 4,652(62.8) | 152(64.4) | 129(60.0) | 45(60.0) | 36(59.0) | 47(78.3) |  |
| Frozen | 1,727(23.3) | 59(25.0) | 60(27.9) | 17(22.7) | 17(27.9) | 7(11.7) |  |
| No transfer | 1,028(13.9) | 25(10.6) | 26(12.1) | 13(17.3) | 8(13.1) | 6(10.0) |  |
| **Stage of transferred embryo** |  |  |  |  |  |  | 0.744 |
| Cleavage stage | 5,475(85.8) | 177(83.9) | 161(85.2) | 53(85.5) | 43(81.1) | 49(90.7) |  |
| Blastocyst stage | 904(14.2) | 34(16.1) | 28(14.8) | 9(14.5) | 10(18.9) | 5(9.3) |  |
| **Number of transferred embryo** |  |  |  |  |  |  | 0.868 |
| one | 1,401(22.0) | 44(20.9) | 45(23.8) | 17(27.4) | 13(24.5) | 11(20.4) |  |
| two | 4,978(78.0) | 167(79.1) | 144(76.2) | 45(72.6) | 40(75.5) | 43(79.6) |  |
| FCP, female chromosomal polymorphism; BMI, body mass index; FSH, follicle stimulating hormone; AFC, antral follicle count; PCOS, polycystic ovarian syndrome; IVF, in vitro fertilization; ICSI, intracytoplasmic sperm injection. | | | | | | | |
| Continuous variables were displayed as the median along with the 25th and 75th percentiles, and their comparisons were conducted using the Kruskal–Wallis test. Categorical variables were presented as the number and percentage, and their comparisons were performed using the Chi-square test. | | | | | | | |
| P values less than 0.050 were shown in bold. | | | | | | | |

| **Table S9 Comparison of baseline characteristics between the control and FCP group in couples undergoing ICSI** | | | |
| --- | --- | --- | --- |
|  | **Control n=3,381** | **FCP group n=304** | ***P* value** |
| **Maternal age, years** | 32.0(29.0~34.0) | 32.0(29.0~35.0) | 0.709 |
| **Paternal age, years** | 33.0(30.0~36.0) | 33.0(30.0~36.0) | 0.994 |
| **Maternal BMI, kg/m2** | 22.0(20.2~24.8) | 21.5(20.0~24.9) | 0.292 |
| **Paternal BMI, kg/m2** | 25.1(22.9~27.8) | 25.0(22.8~28.0) | 0.846 |
| **Basal FSH level** | 6.2(4.8~7.8) | 6.1(4.8~7.6) | 0.734 |
| **Basal E2 level** | 154.0(115.0~199.0) | 152.0(120.0~203.0) | 0.558 |
| **AFC** | 11.0(8.0~15.0) | 10.5(8.0~15.0) | 0.143 |
| **Infertility type** |  |  | 0.267 |
| Primary | 2,372(70.2) | 204(67.1) |  |
| Secondary | 1,009(29.8) | 100(32.9) |  |
| **Tubal factor** |  |  | 0.398 |
| No | 2,724(80.6) | 251(82.6) |  |
| Yes | 657(19.4) | 53(17.4) |  |
| **PCOS** |  |  | 0.250 |
| No | 2,852(84.4) | 264(86.8) |  |
| Yes | 529(15.6) | 40(13.2) |  |
| **Diminished ovarian reserve** |  |  | 0.359 |
| No | 3,072(90.9) | 281(92.4) |  |
| Yes | 309(9.1) | 23(7.6) |  |
| **Endometriosis** |  |  | 0.455 |
| No | 3,142(92.9) | 279(91.8) |  |
| Yes | 239(7.1) | 25(8.2) |  |
| **Other maternal factors** |  |  | 0.076 |
| No | 2,814(83.2) | 265(87.2) |  |
| Yes | 567(16.8) | 39(12.8) |  |
| **Paternal factor** |  |  | 0.208 |
| No | 942(27.9) | 95(31.3) |  |
| Yes | 2,439(72.1) | 209(68.8) |  |
| **Stimulation protocol** |  |  | 0.815 |
| GnRH agonist | 1,234(36.5) | 113(37.2) |  |
| GnRH antagonist | 2,147(63.5) | 191(62.8) |  |
| **Embryo transfer method** |  |  | 0.810 |
| Fresh | 2,117(62.6) | 196(64.5) |  |
| Frozen | 802(23.7) | 68(22.4) |  |
| No transfer | 462(13.7) | 40(13.2) |  |
| **Stage of transferred embryo** |  |  | 0.708 |
| Cleavage stage | 2,566(87.9) | 230(87.1) |  |
| Blastocyst stage | 353(12.1) | 34(12.9) |  |
| **Number of transferred embryo** |  |  | 0.699 |
| one | 590(20.2) | 56(21.2) |  |
| two | 2,329(79.8) | 208(78.8) |  |
| **Type of FCPs** |  |  |  |
| qh+ | NA | 116(38.2) |  |
| pstk+ | NA | 92(30.3) |  |
| inv(9) | NA | 43(14.1) |  |
| multiple | NA | 30(9.9) |  |
| others | NA | 23(7.6) |  |
| FCP, female chromosomal polymorphism; BMI, body mass index; FSH, follicle stimulating hormone; AFC, antral follicle count; PCOS, polycystic ovarian syndrome; IVF, in vitro fertilization; ICSI, intracytoplasmic sperm injection; NA, not applicable. | | | |
| Continuous variables were displayed as the median along with the 25th and 75th percentiles, and their comparisons were conducted using the Mann-Whitney U test. Categorical variables were presented as the number and percentage, and their comparisons were performed using the Chi-square test. | | | |

| **Table S10 Comparison of embryological outcomes between the control and FCP group in couples undergoing ICSI** | | | | |
| --- | --- | --- | --- | --- |
|  | **EMM±SEM** | | **Coefficient (95% CI)** | ***P* value** |
|  | **Control n=3,381** | **FCP group n=304** |  |  |
| **Oocytes retrieved** | 12.8±0.1 | 13.2±0.4 | 0.31(-0.56~1.18) | 0.482 |
| **Metaphase2(M2) oocytes** | 9.9±0.1 | 9.9±0.3 | -0.05(-0.74~0.64) | 0.889 |
| **Oocyte maturation rate (%)** | 78.8±0.3 | 76.0±1.0 | -0.03(-0.05~-0.01) | **0.008** |
| **Normal fertilization rate (%)** | 63.8±0.4 | 60.9±1.4 | -0.03(-0.06~0.00) | 0.051 |
| **Cleavage rate(%)** | 98.5±0.1 | 98.5±0.4 | 0.00(-0.01~0.01) | 0.948 |
| **Transplantable embryo rate (%)** | 58.5±0.6 | 58.5±1.9 | 0.00(-0.04~0.04) | 0.992 |
| FCP, female chromosomal polymorphism; EMM, estimated marginal mean; SEM, standard error of the means; CI, confidence interval | | | | |
| The EMMs, coefficients with their corresponding 95% CIs, and P values were calculated using generalized linear regression models. | | | | |
| P values less than 0.050 were shown in bold. | | | | |

| **Table S11 Comparison of clinical outcomes between the control and FCP group in couples undergoing ICSI^a^** | | | | |
| --- | --- | --- | --- | --- |
|  | **Control** | **FCP group** | **RR (95% CI)** | ***P* value** |
| **Biochemical pregnancy rate** (cases/study subjects (%)) | 1,080/2,919(37.0) | 98/264(37.1) | 1.00(0.85~1.18) | 0.969 |
| **Clinical pregnancy rate** (cases/study subjects (%)) | 901/2,919(30.9) | 77/264(29.2) | 0.95(0.78~1.15) | 0.570 |
| **Miscarriage rate** (cases/study subjects (%)) | 307/901(34.1) | 19/77(24.7) | 0.72(0.49~1.08) | 0.114 |
| **Preterm birth rate** (cases/study subjects (%)) | 86/560(15.4) | 7/52(13.5) | 0.88(0.43~1.79) | 0.718 |
| **Live birth rate** (cases/study subjects (%)) | 558/2,906(19.2) | 52/263(19.8) | 1.03(0.80~1.33) | 0.822 |
| ^a^The number of couples at each stage was depicted in Fig. S3, while the methods employed for calculation were elucidated in the Materials and Methods Section. Couples who were lost to follow-up were excluded from the calculation of the live birth rate. | | | | |
| FCP, female chromosomal polymorphism; RR, risk ratio; CI, confidence interval. | | | | |
| Log-binomial regression models were employed to calculate the RRs with their corresponding 95% CIs and P values. | | | | |

| **Table S12 Comparison of baseline characteristics between the control and FCP subgroups in couples undergoing ICSI** | | | | | | | |
| --- | --- | --- | --- | --- | --- | --- | --- |
|  | **Control n=3,381** | **qh+ n=116** | **pstk+ n=92** | **inv(9) n=43** | **multiple n=30** | **others n=23** | ***P* value** |
| **Maternal age, years** | 32.0(29.0~34.0) | 31.5(29.0~34.0) | 32.0(29.0~35.0) | 31.0(28.0~35.0) | 32.0(29.0~36.0) | 31.0(29.0~33.5) | 0.996 |
| **Paternal age, years** | 33.0(30.0~36.0) | 33.0(30.0~36.0) | 33.5(29.5~36.0) | 33.0(30.0~37.5) | 32.0(29.0~37.0) | 31.0(28.5~36.5) | 0.936 |
| **Maternal BMI, kg/m2** | 22.0(20.2~24.8) | 21.5(20.2~25.0) | 21.5(19.9~24.8) | 22.4(20.7~25.0) | 21.5(19.0~24.8) | 21.6(20.5~25.0) | 0.560 |
| **Paternal BMI, kg/m2** | 25.1(22.9~27.8) | 24.5(22.6~28.4) | 25.3(23.7~27.3) | 24.8(23.3~28.1) | 25.4(23.3~27.4) | 26.4(22.3~28.8) | 0.983 |
| **Basal FSH level** | 6.2(4.8~7.8) | 5.9(4.7~7.8) | 6.1(5.0~7.5) | 6.6(4.9~7.7) | 6.7(6.0~8.5) | 5.5(3.9~6.7) | 0.213 |
| **Basal E2 level** | 154.0(115.0~199.0) | 156.5(120.0~207.0) | 143.5(111.0~207.0) | 138.5(114.0~191.0) | 169.0(128.5~204.9) | 169.0(136.5~198.5) | 0.600 |
| **AFC** | 11.0(8.0~15.0) | 11.5(8.0~15.0) | 10.0(8.0~14.0) | 14.0(9.0~17.0) | 10.0(8.0~14.0) | 11.0(7.0~15.0) | 0.139 |
| **Infertility type** |  |  |  |  |  |  | 0.471 |
| Primary | 2,372(70.2) | 77(66.4) | 64(69.6) | 32(74.4) | 18(60.0) | 13(56.5) |  |
| Secondary | 1,009(29.8) | 39(33.6) | 28(30.4) | 11(25.6) | 12(40.0) | 10(43.5) |  |
| **Tubal factor** |  |  |  |  |  |  | 0.466 |
| No | 2,724(80.6) | 95(81.9) | 75(81.5) | 33(76.7) | 26(86.7) | 22(95.7) |  |
| Yes | 657(19.4) | 21(18.1) | 17(18.5) | 10(23.3) | 4(13.3) | 1(4.3) |  |
| **PCOS** |  |  |  |  |  |  | 0.545 |
| No | 2,852(84.4) | 102(87.9) | 76(82.6) | 37(86.0) | 28(93.3) | 21(91.3) |  |
| Yes | 529(15.6) | 14(12.1) | 16(17.4) | 6(14.0) | 2(6.7) | 2(8.7) |  |
| **Diminished ovarian reserve** |  |  |  |  |  |  | 0.284 |
| No | 3,072(90.9) | 104(89.7) | 88(95.7) | 42(97.7) | 26(86.7) | 21(91.3) |  |
| Yes | 309(9.1) | 12(10.3) | 4(4.3) | 1(2.3) | 4(13.3) | 2(8.7) |  |
| **Endometriosis** |  |  |  |  |  |  | 0.565 |
| No | 3,142(92.9) | 107(92.2) | 85(92.4) | 38(88.4) | 29(96.7) | 20(87.0) |  |
| Yes | 239(7.1) | 9(7.8) | 7(7.6) | 5(11.6) | 1(3.3) | 3(13.0) |  |
| **Other maternal factors** |  |  |  |  |  |  | 0.160 |
| No | 2,814(83.2) | 101(87.1) | 76(82.6) | 40(93.0) | 29(96.7) | 19(82.6) |  |
| Yes | 567(16.8) | 15(12.9) | 16(17.4) | 3(7.0) | 1(3.3) | 4(17.4) |  |
| **Paternal factor** |  |  |  |  |  |  | 0.353 |
| No | 942(27.9) | 32(27.6) | 35(38.0) | 13(30.2) | 7(23.3) | 8(34.8) |  |
| Yes | 2439(72.1) | 84(72.4) | 57(62.0) | 30(69.8) | 23(76.7) | 15(65.2) |  |
| **Stimulation protocol** |  |  |  |  |  |  | 0.876 |
| GnRH agonist | 1,234(36.5) | 41(35.3) | 35(38.0) | 16(37.2) | 14(46.7) | 7(30.4) |  |
| GnRH antagonist | 2,147(63.5) | 75(64.7) | 57(62.0) | 27(62.8) | 16(53.3) | 16(69.6) |  |
| **Embryo transfer method** |  |  |  |  |  |  | 0.999 |
| Fresh | 2,117(62.6) | 73(62.9) | 61(66.3) | 28(65.1) | 18(60.0) | 16(69.6) |  |
| Frozen | 802(23.7) | 28(24.1) | 19(20.7) | 10(23.3) | 7(23.3) | 4(17.4) |  |
| No transfer | 462(13.7) | 15(12.9) | 12(13.0) | 5(11.6) | 5(16.7) | 3(13.0) |  |
| **Stage of transferred embryo** |  |  |  |  |  |  | 0.474 |
| Cleavage stage | 2,566(87.9) | 89(88.1) | 69(86.3) | 34(89.5) | 19(76.0) | 19(95.0) |  |
| Blastocyst stage | 353(12.1) | 12(11.9) | 11(13.8) | 4(10.5) | 6(24.0) | 1(5.0) |  |
| **Number of transferred embryo** |  |  |  |  |  |  | 0.847 |
| one | 590(20.2) | 20(19.8) | 19(23.8) | 6(15.8) | 7(28.0) | 4(20.0) |  |
| two | 2,329(79.8) | 81(80.2) | 61(76.3) | 32(84.2) | 18(72.0) | 16(80.0) |  |
| FCP, female chromosomal polymorphism; BMI, body mass index; FSH, follicle stimulating hormone; AFC, antral follicle count; PCOS, polycystic ovarian syndrome; IVF, in vitro fertilization; ICSI, intracytoplasmic sperm injection. | | | | | | | |
| Continuous variables were displayed as the median along with the 25th and 75th percentiles, and their comparisons were conducted using the Kruskal–Wallis test. Categorical variables were presented as the number and percentage, and their comparisons were performed using the Chi-square test or Fisher exact test. | | | | | | | |

**Supplementary Figures**


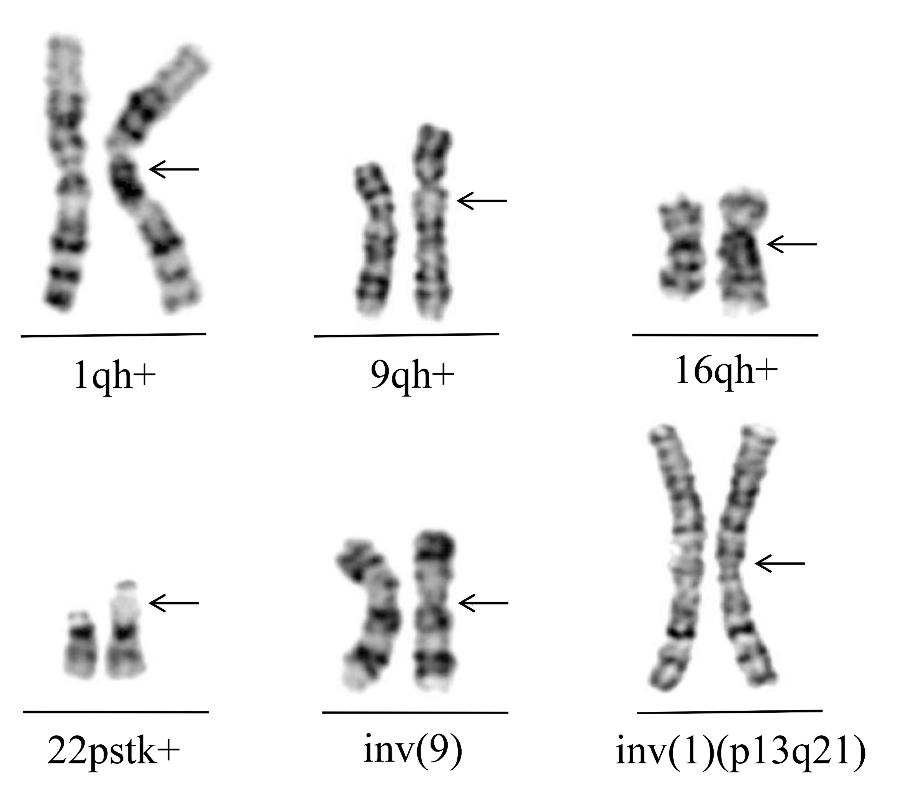


**Fig. S1 Representative images of chromosomal polymorphisms**


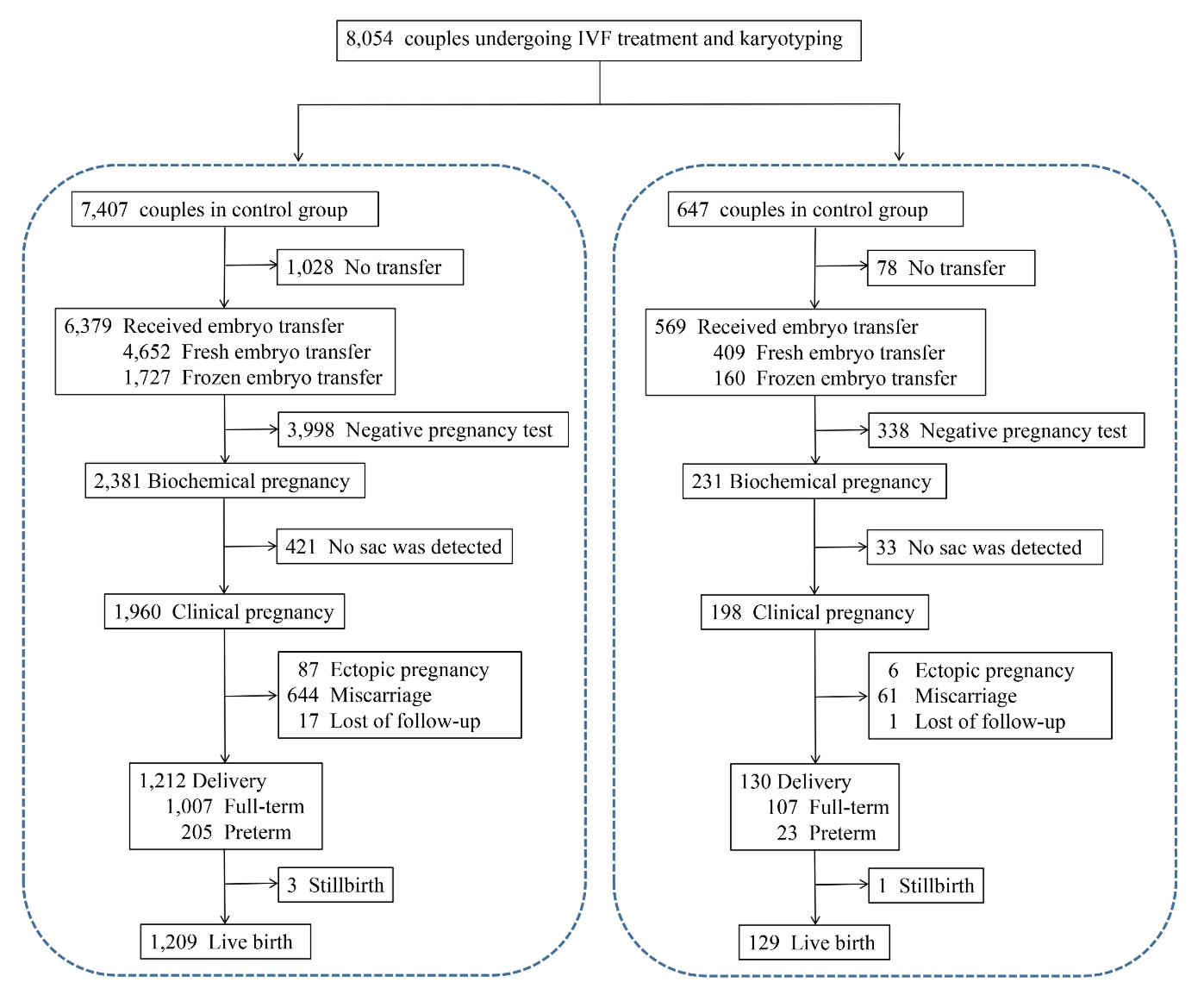


**Fig. S2 Flow chart of participants at each stage of IVF treatment**


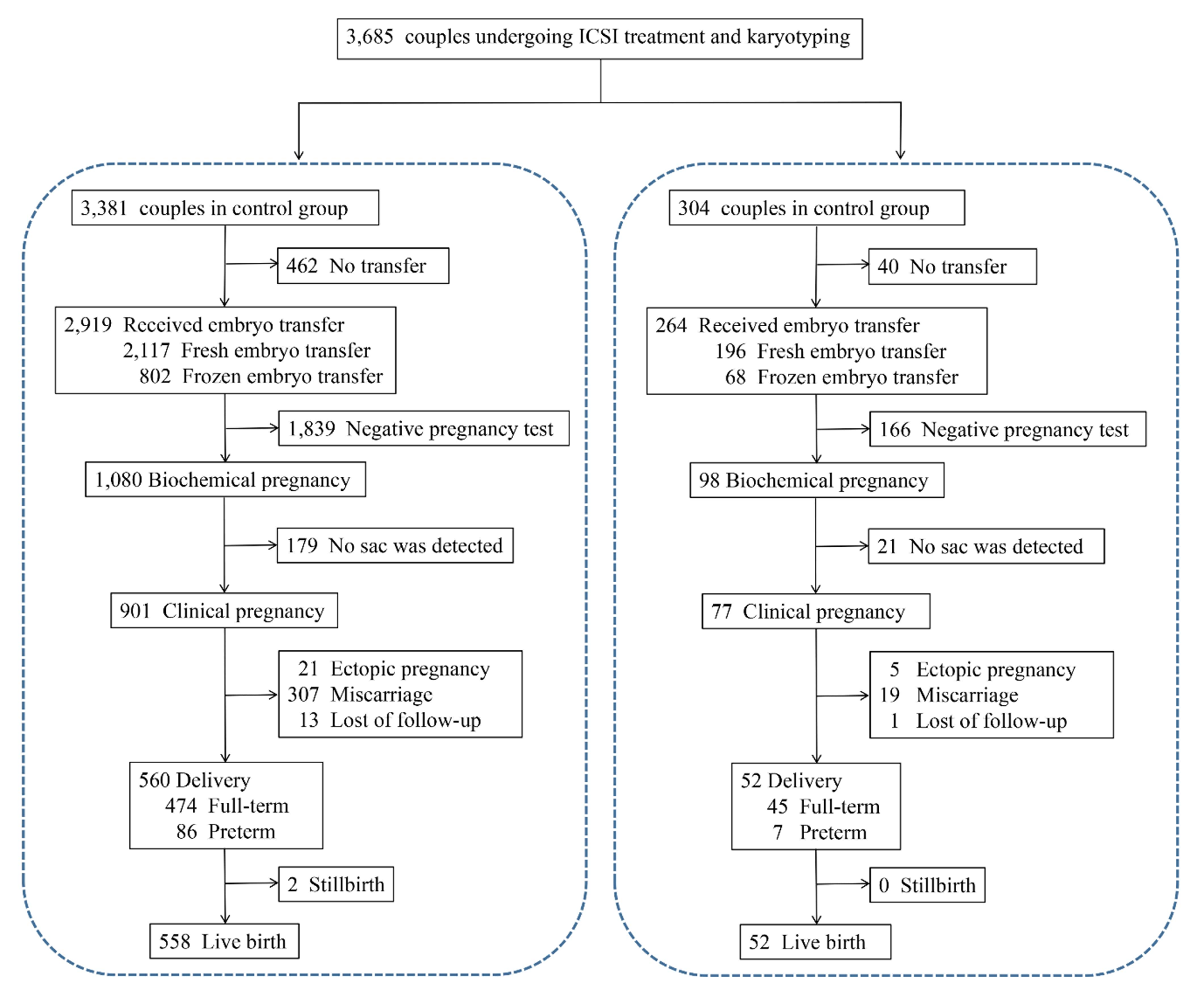


**Fig. S3 Flow chart of participants at each stage of ICSI treatment**
